# THE POWER OF CANCER KNOWLEDGE EXCHANGE AMONG OLDER LATINOS

**DOI:** 10.1093/geroni/igz038.2170

**Published:** 2019-11-08

**Authors:** Iraida V Carrion, Tania Estapé, Malinee Neelamegam, Jane Roberts, Jorge Estapé

**Affiliations:** 1 University of South Florida School of Social Work, Tampa, Florida, United States; 2 FEFOC, Fundacion Contra El Cáncer, Barcelona, Spain, Spain; 3 University of South Florida Sarasota-Manatee, Sarasota, Florida, United States

## Abstract

Prior studies have indicated that older Latinos/as diagnosed with cancer experience social inequalities and other barriers due to their limited English language proficiency and access to health care (Fernández & Morales, 2007). In addition, for Latinos, a cancer diagnosis magnifies health disparities substantially (Gehlert & Colditz, 2011). Despite the impact of the cancer experience, Latinos manifest meaning-based beliefs and coping strategies in dealing with cancer diagnoses (Carrion, Nedjat-Haiem, Macip-Billbe, Black, 2017). However, little is known about older Latinos’ (60 years and older) transmission of knowledge, beliefs, and attitudes to family members and friends. Understanding older Latinos’ advice regarding cancer is essential, given their role in the transmission of knowledge. This study disseminates the latest qualitative findings on older Latinos/as and explores the perspectives shared with family members and friends by non-cancer participants. It explores the types of knowledge, beliefs, attitudes, and advice older Latinos provide to family members and friends about cancer. The data represent individuals without cancer (n=168) residing in the Greater Tampa Bay area. Latinos manifest meaning-based beliefs and coping strategies that assist in dealing with their cancer diagnoses and access to care. Recruitment occurred in community-based settings, with interviews conducted in Spanish and transcribed into English. Qualitative data were analyzed using a constant comparison method and coded in ATLAS.ti to identify emerging themes, including knowledge that a cancer diagnosis is beyond individual control and is in God’s hands, careful choice of a doctor to ensure proper prevention and treatment, and a positive attitude despite the cancer diagnosis.

